# Analysis of Biomarkers and Marginal Bone Loss in Platform-Switched and Nonplatform-Switched Implants: A Randomized Clinical Trial

**DOI:** 10.1155/2022/2603287

**Published:** 2022-05-21

**Authors:** Necla Asli Kocak-Oztug, Gamze Zeynep Adem-Siyli, Orkhan Abishev, Sule Batu, Yegane Guven, Ali Cekici, Aslan Y. Gokbuget, Erhan Firatli, Serdar Cintan

**Affiliations:** ^1^Istanbul University Faculty of Dentistry, Department of Periodontology, Istanbul, Turkey; ^2^Istanbul University Faculty of Dentistry, Department of Biochemistry, Istanbul, Turkey; ^3^Istanbul Kent University Faculty of Dentistry, Department of Biochemistry, Istanbul, Turkey; ^4^Department of Periodontology, PGG Private Practice, Istanbul, Turkey; ^5^Nisantasi University Faculty of Dentistry, Department of Periodontology, Istanbul, Turkey

## Abstract

**Objectives:**

To compare the peri-implant crevicular fluid (PICF) biomarker levels, peri-implant status, and marginal bone level (MBL) differences of implants restored with randomly assigned nonplatform-switched (NPS) or platform-switched (PS) abutments.

**Methods:**

Ninety-four implants in 27 subjects were included in this study. Receptor activator of nuclear factor kappa-B ligand (RANKL), osteoprotegerin (OPG), interleukin-1*β* (IL-1*β*), monocyte chemotactic protein-1 (MCP-1) levels in PICF, peri-implant health, and the change in the MBL were evaluated at the time of restoration (*T*_1_) and after 12 months (*T*_2_).

**Results:**

The IL-1*β* levels decreased and the RANKL, OPG, and MCP-1 levels increased from *T*_1_ to *T*_2_ (*P* < 0.05) in both groups. RANKL/OPG ratio at *T*_1_, MCP-1 levels at *T*_2_, and the MCP-1 change from *T*_1_ to *T*_2_ were lower in the PS group than in the NPS group (*P* < 0.05). MBL change was lower (0.51 ± 0.31 mm) in the PS group than that (0.75 ± 0.29 mm) in the NPS group at *T*_2_ (*P* < 0.001). Peri-implant health status between the study groups was negligible.

**Conclusion:**

PS was superior to NPS regarding the preservation of MBL. Higher MCP-1 levels, altered RANKL/OPG ratio, and lower OPG levels in the NPS group could be associated with subclinical peri-implant bone remodeling.

## 1. Introduction

Maintaining peri-implant marginal bone level (MBL) is the key factor for success in implant dentistry. During the first year of function, with a stable physiological state, the marginal bone around the implant is remodelled with a vertical interval from 1.5 mm to 3 mm [1, 2]. There is some clinical evidence that this bone resorption could be reduced by attaching a platform abutment with a narrower diameter to the implant, which is known as the platform-switching (PS) concept [3–6]. However, the mechanism for this reduction in bone loss around a PS implant is not fully resolved [7]. The concept of PS might be understood about inhibiting or diminishing inflammatory mediators (e.g., cytokines, bone biomarkers, and chemokines) that may be released by peri-implant tissue cells depending on the type of abutment selected. Some cytokines in peri-implant crevicular fluid (PICF) have been indicated as potentially legitimate diagnostic biomarkers of bone resorption in the presence of peri-implant disease [8]. Even so, no biomarker that can display early bone loss around a healthy peri-implant after loading has been clarified.

Recently, there has been enthusiasm to identify the association between crestal bone resorption around implants and the key biomarkers of osteoclastogenesis in PICF [9, 10]. Osteoclastogenesis is mainly regulated by the following biomarkers: receptor activator of nuclear factor kappa-B (RANK), RANK's ligand (RANKL), and osteoprotegerin (OPG). Elevated PICF RANK or RANKL levels and higher RANKL/OPG ratios have been shown to be related to greater bone resorption around implants. Conversely, increased PICF OPG levels and decreased ratios of RANKL/OPG are associated with healthy peri-implant areas [11–13]. Interleukin-1 beta (IL-1*β*) and monocyte chemotactic protein-1 (MCP-1) also play important roles in bone mechanism [9, 12, 14]. Notably, elevated PICF IL-1*β* levels in peri-implant diseases have been reported [9, 12]. Moreover, MCP-1 has been extensively studied especially in bone cancer research. The conclusion of these studies is that even in the absence of RANKL, MCP-1 may induce osteoclastogenesis by acting on monocytes to induce bone resorption, indicating that MCP-1 is a valuable biomarker for bone remodeling [10, 15].

To date, human trials heavily evaluated biomarker levels in PICF and their relationship with peri-implant disease [9, 11, 16]. However, studies focusing on biochemical analysis of PS with PICF biomarkers are limited [4, 17]. This study is aimed at addressing the abovementioned research gap, focusing on biochemically investigating the association between marginal bone loss, peri-implant status, and RANKL, OPG, IL-1*β*, and MCP-1 levels in PICF around PS and nonplatform-switched (NPS) implants in the first year of function. The interrelation between clinical, radiographic, and biochemical analyses may help researchers identify the mechanism of the PS concept and evaluate the effects of abutment modification on the host's local tissue response. In addition, analysis of PICF biomarkers may assist clinicians in detecting the early signals of peri-implant disease and coming up with a corrective treatment.

## 2. Materials and Methods

### 2.1. Patient Selection and Study Design

In this study, implants of all subjects were randomly allocated to the NPS group or PS group. Assignment of the implants to the groups using a split-mouth design was performed with a software (http://www.randomizer.org/) by a specialist apart from the surgeon and was saved in a sealed envelope until surgery. A balanced random permuted block approach was followed, ensuring that roughly equal numbers of participants were allocated to all the comparison groups at any point in the trial. Patients were informed about the procedure but were blinded which implant was belonged to which group [18–20]. The study population was selected from patients who applied for implant therapy at the Department of Periodontology, Istanbul University (IU), after clinical and radiographic examinations from 2014 to 2018. Formal consent was acquired from all patients. The protocol was endorsed by the Ethics Commission of IU (No: 2013/1069) and guided in accordance with the Helsinki Declaration. This study was prepared according to the CONSORT guidelines, and the clinical trial registration number is TCTR20210721008.

In this study, the inclusion criteria were (1) in need of no less than two implants in the posterior area, (2) full-mouth plaque index and full-mouth bleeding score ≤ 25%, (3) sites without acute infection or bone defects, (4) ≥7 mm width and ≥9 mm height of bone, (5) presence of ≥2 mm width peri-implant keratinized tissue, (6) nonsmoker, and (7) aged 30–70 years. Exclusion criteria were (1) untreated periodontal disease and (2) systemic conditions and/or any medication(s) that could influence soft and hard tissue healing mechanisms.

The size (3.8 or 4.3 mm diameter and 9, 11, or 13 mm length) of the implants (Camlog Biotechnologies AG, Basel, Switzerland) were selected according to the amount of available bone. The abutments in the PS group had a similar design as the NPS ones except inward positioning of the implant/abutment junction. This position created a 0.3 mm platform all around PS abutment and the implant neck [5, 18–20]. To minimize confounding factors, equivalency in implant height and width and the placement zone were ensured between the groups. It was ensured that the distance between the adjacent teeth and the implants was 1.5 mm, the distance between the implants was at least 3 mm, and at least 1.5 mm of solid bone around the implants was maintained [5]. The same experienced surgeon placed the implants via one-stage surgery at the crestal bone level (minimum torque of 30 N-cm). PS or NPS healing abutments were placed in the PS and NPS groups according to random assignment right after implant insertion to achieve transgingival healing. Patients were prescribed 1000 mg of antibiotics (amoxicillin 875 mg plus clavulanic acid 125 mg), 550 mg of nonsteroidal anti-inflammatory agent (naproxen sodium 550 mg), and 0.2% chlorhexidine mouth rinse twice a day for one week. Twelve weeks later, the permanent supra structures were created by a single prosthodontist. Implant impressions were made using polyvinylsiloxane material in a single-step procedure using the manufacturer's measurement headers and analogues for implant-based prosthetic application for both study groups. Abutments were torqued with 30 N-cm using a ratchet as recommended by the manufacturer. Implant-supported fixed prostheses were cemented, and any residual cement was cleaned immediately using floss and scalers [18, 21].

### 2.2. Clinical and Radiographic Analyses

**Figure 1:**
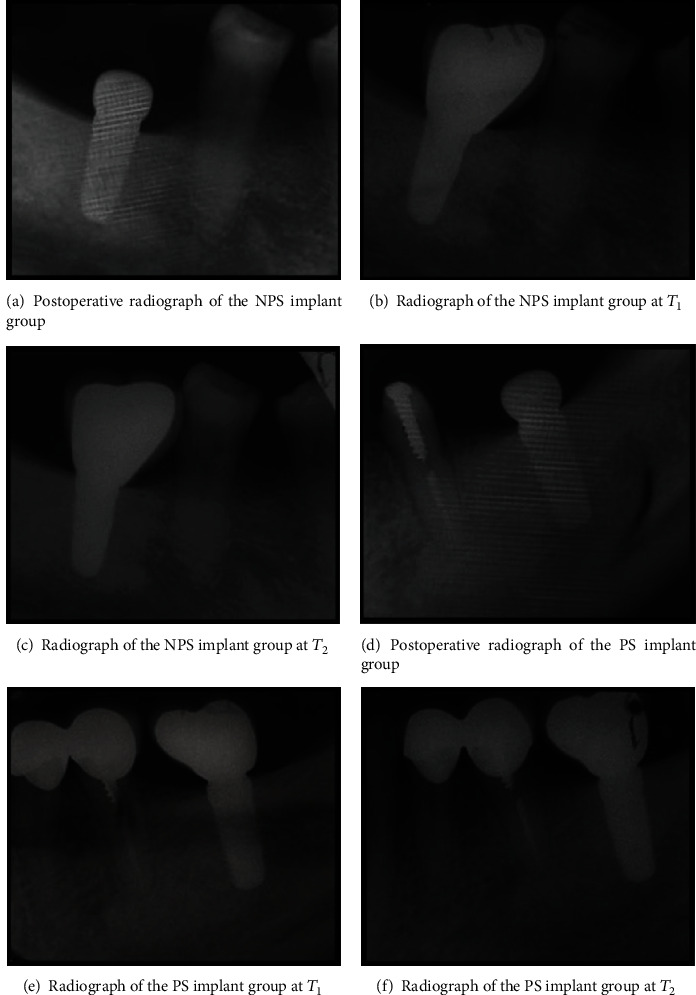
Representative radiographs of study groups: (a–c) nonplatform-switched group and (d–f) platform-switched group.

**Figure 2:**
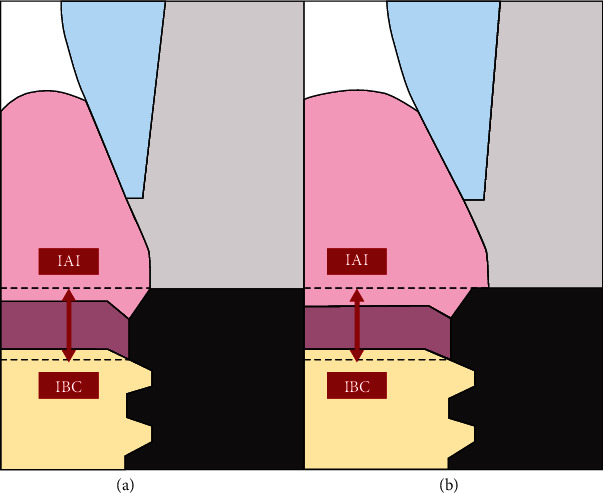
Representation of implant abutment interface (IAI) and bone implant connection (IBC) for (a) nonplatform-switched and (b) platform-switched implants.

To observe the clinical health status around the implants, the bleeding on probing (BOP), modified plaque index (mPI), and probing pocket depth (PPD) were recorded by the same examiner (who was not the implant surgeon) 12 weeks postsurgery right after of loading (*T*_1_) and 12 months postloading (*T*_2_). All measurements were taken from four implant sites, i.e., vestibulo-mesial, vestibulo-distal, palatino-mesial, and palatino-distal [22–25]. Radiographs were obtained from each implant right after implant surgery and at *T*_1_ and *T*_2_ using standardized periapical radiographs with the long cone paralleling technique by a single radiology specialist. To secure parallelism and standardization of all radiographs, an occlusal jig was prepared for each patient. These radiographs were used to calculate MBL values from the mesial and distal sides of the implants at *T*_1_ and *T*_2_ using ImageJ 1.52 (Wisconsin, USA) [25–27] (Figure 1). The junction between the implant-abutment interface (IAI) was used as the reference, and the distance between the IAI and the coronal bone-implant connection (IBC) was measured (Figure 2). To prevent bias and ensure excellent reliability, the measurements were obtained by the same calibrated examiner (*R* = 0.937). To ensure that the MBL measurements were not affected by scale and distortion, the computed distance measurements were corrected by applying the scale (measurements and actual distances) to the implant width and length. The mean value of the mesial and distal measurements was used to represent the crestal bone loss for all implants.

### 2.3. PICF Sampling and Biochemical Analysis

Before sampling, implant sites were isolated using cotton rolls and air-dried to eliminate salivary contamination. Paper strips (PerioPaper; Oraflow, NY, USA) were placed 2-3 mm into the peri-implant crevice with forceps for 30 seconds at the vestibulo-mesial and vestibulo-distal sites of each implant [28]. In case of blood contamination, the strip was discarded. For each implant, the strips were pooled in a coded Eppendorf tube, weighed, and kept at −80°C until biochemical analysis. To evaluate the RANKL, OPG, MCP-1, and IL-1*β* levels, PICF samples were analyzed using enzyme-linked immunoassay (ELISA) in the Department of Biochemistry, IU. Briefly, the strips were removed from the −80°C storage and dissolved for one hour at room temperature. Subsequently, 200 *μ*l of diluent buffer (pH 7.4; phosphate-buffered saline) was added. Impregnated PICF samples were passed through the diluent solution as described previously [29]. The IL-1*β*, MCP-1 (Diaclone, Besancon, France), RANKL, and OPG (YH Biosearch Laboratory, Shanghai, China) ELISA kits were executed according to the manufacturer's guidelines [8, 21, 29, 30].

### 2.4. Statistical Analysis

Power analysis was conducted for the number of samples required (G^∗^ Power version 3.1.7). The study power was expressed as 1-*β* (*β* indicating the probability of type II error). Based on the group differences of RANKL/OPG ratios in the article of Guncu et al. the effect size (*d*) was 0.712 in the calculation made to obtain 80% power at the *α* = 0.05 level [12]. Accordingly, it was calculated that there should be at least 32 implants in every group. We enrolled 47 implants in each group to allow for possible loss of sample size.

Statistical analyses were conducted using SPSS V 22.0 (IBM Corp., NY, USA). The distribution of data was evaluated for normality using the Shapiro-Wilks test. Student's *t*-test was used to compare normally distributed data, and the Mann–Whitney *U* test was performed to compare nonnormally distributed data among the groups. The paired-sample *t*-test was performed for intragroup comparison of normally distributed data and the Wilcoxon sign test was used for intragroup comparison of non-normally distributed data. The chi-squared test and continuity (Yates) correction were used to compare qualitative data. The quantitative data were demonstrated as mean ± standard deviation (SD). *P* < 0.05 was the level of statistical significance.

## 3. Results

**Figure 3:**
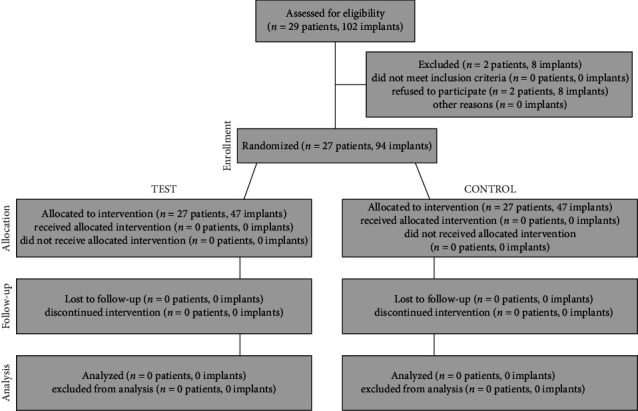
CONSORT flow diagram of the clinical trial.

A CONSORT flow diagram is presented in Figure 3.

### 3.1. Demographic Data, Peri-Implant Status, and MBL Changes

Twenty-seven patients (11 men, 16 women) of mean age 45.63 ± 10.67 years were included in this trial. Ninety-four implants, each supporting a fixed prosthesis, were randomly assigned to the NPS group (*n* = 47) or the PS group (*n* = 47). The PS and NPS groups exhibited no statistically significant variation in the distribution of jaw type, implant diameter, or implant length (*P* > 0.05, Table 1).

The mPI and BO*P* values increased significantly in both groups between *T*_1_ and *T*_2_ (*P* < 0.01), and there was no statistically significant difference for both parameters at *T*_1_ or *T*_2_ or in the changes from *T*_1_ to *T*_2_ between the groups (*P* > 0.05, Table 2). There was no statistically significant difference in PPD values at *T*_1_ between the groups (*P* > 0.05). At *T*_2_, PPD values in the PS group were significantly lower than those in the NPS group (*P* < 0.05). In both groups, the rise in PPD values between *T*_1_ and *T*_2_ was statistically significant (*P* < 0.01); however, the change from *T*_1_ to *T*_2_ between the two groups was not statistically significant (*P* > 0.05, Table 3).

There was no significant difference in MBLs between the PS and NPS groups at *T*_1_ (*P* > 0.05). In both groups, the increase in bone loss that occurred between *T*_1_ and *T*_2_ was statistically significant (*P* < 0.01, Table 3). The mean bone loss in the PS group at *T*_2_ was significantly lower than that in the NPS group (*P* < 0.01). The bone loss seen in the NPS group between *T*_1_ and *T*_2_ was significantly greater than that in the PS group (*P* < 0.01, Table 3).

### 3.2. Biochemical Findings

The OPG levels were significantly higher in the PS group than in the NPS group (*P* < 0.05), and the RANKL levels were not statistically significant between the two groups (*P* > 0.05) at *T*_1_. There was no significant difference in the RANKL or OPG level between the groups at *T*_2_ (*P* > 0.05, Table 4). In both groups, the increase in the levels of RANKL and OPG from *T*_1_ to *T*_2_ was statistically significant (*P* < 0.01). For both markers, these differences were not statistically significant between the two groups (*P* > 0.05, Table 5). The RANKL/OPG ratios were significantly lower in the PS than in the NPS group at *T*_1_ (*P* < 0.05) and not statistically significant between the groups at *T*_2_ (*P* > 0.05, Table 4). The RANKL/OPG ratio significantly decreased in both groups from *T*_1_ to *T*_2_ (*P* < 0.05), and these differences were not statistically significant between the two groups (*P* > 0.05, Table 5).

At *T*_1_, the MCP-1 levels were lower in the PS group than in the NPS group, but the difference was not statistically significant (*P* > 0.05, Table 4). In both groups, the increases in the MCP-1 levels from *T*_1_ to *T*_2_ were statistically significant (*P* < 0.05 for the PS group, *P* < 0.01 for the NPS group; Table 5). The MCP-1 levels at *T*_2_ and the difference in MCP-1 levels between the two time points in the NPS group were significantly higher than those in the PS group (*P* < 0.05, Tables 4 and 5).

At *T*_1_, the IL-1*β* levels were lower in the PS group than in the NPS group, but the difference was not statistically significant (*P* > 0.05, Table 4). At *T*_2_, the IL-1*β* levels were significantly lower in the PS group than in the NPS group (*P* < 0.05, Table 4). In both groups, there was a statistically significant decrease in IL-1*β* levels between *T*_1_ and *T*_2_ (*P* < 0.01), and the change in IL-1*β* levels was not statistically significant between the groups (*P* > 0.05, Table 5).

## 4. Discussion

The present study evaluated the change in MBL, peri-implant status, and biomarkers in PCIF between PS and NPS abutment-restored implants immediately after loading and 1-year postloading. To our knowledge, there is not enough data in the literature to explain the connection of marginal bone loss with biochemical markers comparing PS and NPS implants [4, 17, 26].

Investigating biomarkers in PICF remain an evolving subject of research, and relevant studies have usually centered on examining biomarkers around implants with peri-implant disease and those around healthy implants [10, 11, 31]. The IAI in NPS implants may lead to inflammation throughout remodeling after loading due to the position of microgap and stress adjacent to the bone may shift the equilibrium of biomarkers, which may cause bone resorption [3].

Proinflammatory biomarkers, such as IL-1*β*, control inflammation, wound activity, and prostaglandin E_2_ synthesis, which play a crucial role in the onset of hard tissue destruction [31]. However, the real control on the local bone integrity depends on the crosstalk between the activity of RANKL and OPG [32]. Recently, MCP-1 has also been shown to induce osteoclastogenesis, even in the absence of RANKL [10, 15, 33]. Therefore, in this clinical trial, we analyzed the levels of IL-1*β*, RANKL, OPG as the RANKL antagonist, and MCP-1 in the PICF.

The balance between the activity of RANKL and OPG is the key factor to maintain the integrity of hard tissues, and an imbalance between RANKL and OPG is associated with the progression of bone resorption [9, 33]. Furthermore, a recent review exploring the studies of biomarkers in PICF revealed that RANKL and OPG might be the key biomarkers to understand the mechanisms of bone loss around implants [11]. In this study, the climb in the levels of RANKL and OPG in both groups over time may indicate the increase in bone activity after loading. The OPG levels were lower in the NPS group in both time points. This difference reached a statistical significance at *T*_1_ whereas not at *T*_2_. The RANKL levels were lower in the PS group at all time points, but this difference was not statistically detectable. Even the individual bone biomarkers increased over time, the ratio of RANKL/OPG exhibited a descending trend during our follow-up for both groups. These results may suggest that bone destruction commenced slower in the PS group than the NPS group after loading at *T*_1_ and that less bone destruction occurred in the PS group during the 12-month follow-up period. These results could also indicate that, at the end of one year, there was no significant difference regarding bone production between the groups. However, to utterly understand in which time interval the RANKL or OPG levels are increasing, further studies are needed. For example, a recent clinical trial by Cheng et al. investigated the effects of PS on soft tissue response after surgery. This trial found that OPG and IL-1*β* levels decreased over the six weeks follow-up period without significant difference between the groups [4].

According to the literature, IL-1*β* is secreted locally during an inflammatory state, accelerating inflammatory reactions with other factors involved in the immune response [28]. Furthermore, there is some evidence suggesting that IL-1*β* is the main proinflammatory biomarker in active peri-implantitis regions; thus, it can be used as an early indicator of peri-implant disease [34]. In this study, the IL-1*β* values at *T*_2_ were significantly higher than those detected at *T*_1_ in both study groups. Given that there was no clinical evidence of inflammation at 12 months, the increase in IL-1*β* levels may be related to the remodeling process. Additionally, after one year of loading, the IL-1*β* levels were significantly higher in the NPS group, indicating that less bone resorption occurred around the PS implants.

Nowzari et al. examined IL-1*β*, RANKL, TNF-alpha, IL-6, and IL-8 levels in their studies involving healthy teeth and healthy implants and reported that these biomarkers were seen more often around healthy implants than healthy teeth, even in the absence of inflammation [31]. In another study, Kajele and Mehta evaluated the PICF around implants after loading and observed elevated IL-1*β* levels after 12 months. These results showed a similar increase in IL-1*β* as in our study. However, unlike ours, this study was based on single implant treatments for single-tooth deficiencies to compare IL-1*β* levels with clinical measurements [16].

MCP-1 is a prime mediator in the synchronization of the inflammatory response in periodontal tissues and is currently popular in animal studies conducted in the cancer field [15]. Interestingly, this study's results revealed significant increases in MCP-1 levels in both groups with time, which can be interpreted as continuing bone activity and remodeling. Additionally, MCP-1 levels were significantly higher at *T*_2_ in the NPS group than in the PS group, suggesting elevated bone resorption in that group.

Irshad et al. found that the MCP-1 level was higher in PICF from peri-implantitis specimens than healthy specimens [9]. Emecen et al. compared MCP-1 levels in PICF and gingival crevicular fluid (GICF). The authors found that individuals having osseointegrated healthy implants showed no differences in MCP-1 levels between PICF and GICF [21]. These studies, consistent with findings we obtained in this study, support the association of MCP-1 with bone destruction. However, there is currently insufficient data in the PS clinical trials to make an adequate comparison regarding relevant biomarkers in this clinical trial.

The use of clinical periodontal parameters for evaluating implant health are insufficient due to the differences between peri-implant and periodontal tissues. Bleeding on probing was shown to be a negative index of health rather than a sign of disease. It does not provide explicit data regarding the activity and path of present disease [35]. Moreover, peri-implant tissue is more prone to probing pressure, which may impair both bleeding and probing depth values. Healthy peri-implant probing depths are higher than periodontal probing depths and range from 2 to 6 mm [34]. Noteworthy, none of the PPD values exceeded 4 mm in our study. In addition, there was no statistically significant difference observed in the PPDs at *T*_1_ between the PS and NPS groups. The PS group's PPDs at *T*_2_ were significantly lower than those of the NPS group. Although this was interpreted as a lower depth of pocket around the PS implants at the end of one year, it should also be considered that PPDs around the implants do not provide as much objective information as the PPDs around teeth.

In our study, the mPI and BOP levels increased significantly during the first year of observation for both groups, but no peri-implant disease was observed, and the increased levels were not statistically significant in the group comparisons. Given that the prosthetic restorations have just been completed at *T*_1_, it could be considered that this surface later provided an additional surface area for plaque retention, which suggests that an increase in plaque retention (as indicated by the increased mPI) may have induced the increase in the BOP.

Chien et al. reported the effects of PS on PICF content during early wound healing after healing abutments were inserted. Contrary to our results, their PS and NPS groups showed a drop in all clinical indices over a 6-week period. These divergent results may reflect the difference in follow-up time between studies and that measurements were recorded immediately after loading in our study [17].

Canullo et al. observed the PS implants for 10 consecutive years with different trials using long cone paralleling radiography. This trial group observed that PS implants were in favor of protecting peri-implant bone and showed less soft tissue shrinkage [25–27, 36]. Like these trials, in this study, radiographs were taken with the long cone paralleling technique to measure peri-implant bone loss. This method was chosen because it is reproducible, straightforward to execute, and commonly used in the literature [36]. On the other hand, this radiography technique only allows visualization of mesial and distal bone, and it is not possible to evaluate the condition of the lingual or buccal bone. This limitation is evident in almost all similar studies reported to date [36–38].

The current study's results revealed that although the survival rate was the same for both abutment types, the PS group exhibited greater bone maintenance during the first year than the NPS group. In accordance with our study, Wang et al. found that the difference in the change in marginal bone level between the PS and NPS implants was significant in favor of the PS implants. In their study, periapical radiographs were also acquired using the long cone paralleling technique. In our study, the change in vertical bone level was 0.75 ± 0.29 mm in the NPS group and 0.51 ± 0.31 mm in the PS group, whereas in the study performed by Wang et al., the change in bone level was −0.04 ± 0.08 mm in the PS group and −0.19 ± 0.16 mm in the control group. This dissimilarity between the measurements may be due to the difference in the methods used to analyze the radiographs [37].

Wagenberg et al. performed a retrospective study of 94 PS implants that were followed for 11 years. In agreement with our findings, although there was no significant difference in success between PS and NPS implants, less bone loss was observed with the PS implants [39].

As in the present study, Gültekin et al. found more bone loss in NPS implants after one year of loading. They also found that a large part of the difference in bone levels occurred within the first six months for both study groups. The authors argued that the remodeling capacity of the bone could be stimulated following implant loading [18]. However, Crespi et al. found no difference in bone loss between their PS and NPS groups during the first year of function in contrast with our findings in this study [40].

## 5. Conclusion

Within the due limits of this clinical trial, PS implants have a significant effect on peri-implant bone preservation throughout the first year of function. Higher MCP-1 levels, altered RANKL/OPG ratio, and lower OPG levels in the NPS group may lead to greater bone destruction in the NPS group. The knowledge of the clinical, radiographic, and biochemical aspects of PS implants can assist clinicians in improving their implant choices. Further studies are needed to resolve the biochemical behavior of the hard tissue around PS implants.

## Figures and Tables

**Table 1:** Distribution of implant placement, diameter, and length in both groups.

		PS, *n* (%)		NPS, *n* (%)	*P* (*ø*)
Jaw type	Maxilla	24	(51.1)	23(48.9)	0.837
	Mandible	23	(48.9)	24 (51.1)	
Implant width (mm)	3.80	31	(66.0)	32 (68.1)	0.826
	4.30	16	(34.0)	15 (31.9)	
Implant length (mm)	9	11	(23.4)	12 (25.5)	0.796
	11	29	(61.7)	26 (55.3)	
	13	7 (14.9)		9 (19.2)	

Comparison of the mean values between the groups was performed using chi-squared test and continuity (Yates) correction ø, *P* > 0.05.

**Table 2:** Comparison of mPI and BOP between the PS and NPS groups.

	mPI			BOP		
	PS	NPS	*P* (*α*)	PS	NPS	*P* (*α*)
*T*_1_	0.25 ± 0.24	0.26 ± 0.24	0.767	0.17 ± 0.21	0.14 ± 0.2	0.168
*T*_2_	0.62 ± 0.34	0.58 ± 0.3	0.511	0.54 ± 0.37	0.51 ± 0.35	0.655
*P* (*β*)	0.001^∗∗^	0.001^∗∗^	—	0.001^∗∗^	0.001^∗∗^	—
Difference	0.37 ± 0.28	0.32 ± 0.33	0.332	0.37 ± 0.37	0.38 ± 0.32	0.834

Comparison of the values between the groups was performed using Mann–Whitney *U* test *α*, *P* > 0.05; comparison of the change from *T*_1_ to *T*_2_ between the groups was performed by using Wilcoxon sign test *β*, ^∗∗^*P* < 0.01.

**Table 3:** Comparison of PPD and MBL between the PS and NPS groups.

	PPD			MBL		
	PS	NPS	*P* (*γ*)	PS	NPS	*P* (*γ*)
*T*_1_	2.19 ± 0.69	2.42 ± 0.76	0.130	0.46 ± 0.27	0.54 ± 0.27	0.168
*T*_2_	2.54 ± 0.78	2.91 ± 0.86	0.036^∗^	0.97 ± 0.33	1.29 ± 0.33	0.001^∗∗^
*P* (*Φ*)	0.001^∗∗^	0.001^∗∗^	—	0.001^∗∗^	0.001^∗∗^	—
Difference	0.35 ± 0.38	0.48 ± 0.52	0.163	0.51 ± 0.31	0.75 ± 0.29	0.001^∗∗^

Comparison of the values between the groups was performed using Student's *t*-test *γ*, ^∗^*P* < 0.05, ^∗∗^*P* < 0.01; comparison of the change from *T*_1_ to *T*_2_ between the groups was performed by using paired-sample *t*-test *Φ*, ^∗∗^*P* < 0.01.

**Table 4:** Biomarker levels in PICF for both groups.

	PS	NPS	*P* (†)
	Mean ± SD	Mean ± SD	
RANKL at *T*_1_	129.01 ± 22.28 ng/ml	132.55 ± 20.22 ng/ml	0.422
RANKL at *T*_2_	156.48 ± 22.91 ng/ml	159.77 ± 27.84 ng/ml	0.532
OPG at *T*_1_	1.39 ± 0.21 ng/ml	1.29 ± 0.21 ng/ml	0.025^∗^
OPG at *T*_2_	1.67 ± 0.19 ng/ml	1.62 ± 0.22 ng/ml	0.236
RANKL/OPG at *T*_1_	95.12 ± 21.74	106.35 ± 29.5	0.038^∗^
RANKL/OPG at *T*_2_	94.78 ± 17.83	100.36 ± 23.44	0.197
MCP-1 at *T*_1_	12.04 ± 1.54 pg/ml	12.13 ± 1.27 pg/ml	0.740
MCP-1 at *T*_2_	12.76 ± 1.68 pg/ml	13.77 ± 1.81 pg/ml	0.006^∗^
IL-1*β* at *T*_1_	69.14 ± 22.84 pg/ml	76.05 ± 19.19 pg/ml	0.116
IL-1*β* at *T*_2_	51.05 ± 20.69 pg/ml	61.49 ± 21.66 pg/ml	0.019^∗^

Comparison of the values between the groups was performed using Student's *t*-test †, ^∗^*P* < 0.05.

**Table 5:** Changes in PICF biomarker levels for both groups.

	Difference	*P* (§)
RANKL PS	27.47 ± 32.38 ng/ml	0.001^∗∗^
RANKL NPS	27.23 ± 37.37 ng/ml	0.001^∗∗^
*P* (¥)	0.973	
OPG PS	0.29 ± 0.22 ng/ml	0.001^∗∗^
OPG NPS	0.33 ± 0.29 ng/ml	0.001^∗∗^
*P* (¥)	0.364	
RANKL/OPG PS	−0.34 ± 27.18	0.932
RANKL/OPG NPS	−5.99 ± 39.37	0.302
*P* (¥)	0.42	
IL-1*β* PS	−18.09 ± 30.62 pg/ml	0.001^∗∗^
IL-1*β* NPS	−14.56 ± 19.43 pg/ml	0.001^∗∗^
*P* (¥)	0.506	
MCP-1 PS	0.72 ± 2.27 pg/ml	0.034^∗^
MCP-1 NPS	1.64 ± 2.06 pg/ml	0.001^∗∗^
*P* (¥)	0.044^∗^	

Comparison of the change in the values from *T*_1_ to *T*_2_ in the groups was performed by using paired sample *t*-test §, ^∗^*P* < 0.05, ^∗∗^*P* < 0.01; comparison of the change in the values ratio from *T*_1_ to *T*_2_ between the groups was performed by using Student's *t*-test ¥, ^∗^*P* < 0.05.

## Data Availability

The data used to assist the results of this study are included within the article.

## Supplementary Materials

Supplementary Materials CONSORT 2010 checklist.
